# siRNA knockdown of GPR18 receptors in BV-2 microglia attenuates *N*-arachidonoyl glycine-induced cell migration

**DOI:** 10.1186/1750-2187-7-10

**Published:** 2012-07-26

**Authors:** Douglas McHugh, James Wager-Miller, Jeremy Page, Heather B Bradshaw

**Affiliations:** 1 Department of Psychological and Brain Sciences, Indiana University, Bloomington, IN, 47405, USA

**Keywords:** GPR18, NAGly, Abnormal cannabidiol receptor, Cellular migration, Endocannabinoid, Microglia

## Abstract

**Background:**

Neurons are known to employ the endogenous cannabinoid system to communicate with other cells of the CNS. Endocannabioid signaling recruits microglia toward neurons by engaging cannabinoid CB_2_ and abnormal cannabidiol (Abn-CBD) receptors. The Abn-CBD receptor is a prominent atypical cannabinoid receptor that had been discriminated by means of various pharmacological and genetic tools but remained to be identified at the molecular level. We recently introduced *N*-arachidonoyl glycine (NAGly) signaling via GPR18 receptors as an important novel signaling mechanism in microglial-neuronal communication. NAGly is an endogenous, enzymatically oxygenated metabolite of the endocannabinoid *N*-arachidonoyl ethanolamide (AEA). Our recent studies support strongly two hypotheses; first that NAGly initiates directed microglial migration in the CNS through activation of GPR18, and second that GPR18 is the Abn-CBD receptor. Here we present siRNA knockdown data in further support of these hypotheses.

**Findings:**

A GPR18-targetting siRNA pSUPER G418 GFP cDNA plasmid was created and transfected into BV-2 microglia. Successfully transfected GFP^+^ GPR18 siRNA BV-2 microglia displayed reduced GPR18 mRNA levels and immunocytochemical staining. Cell migration induced by 1 μM concentrations of NAGly, O-1602 and Abn-CBD were significantly attenuated in GFP^+^ cells.

**Conclusions:**

Our data provide definitive evidence that these compounds, characteristic of Abn-CBD receptor pharmacology, are acting via GPR18 in BV-2 microglia. A fuller understanding of the hitherto unidentified cannabinoid receptors such as GPR18; their molecular interactions with endogenous ligands; and how phytocannabinoids influence their signaling is vital if we are to comprehensively assess the function of the endogenous cannabinoid signaling system in human health and disease.

## Findings

### Background and significance

**Figure 1:**
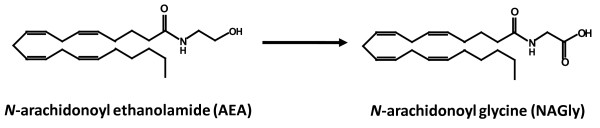
**Chemical structures of AEA and NAGly.** The chemical structures of *N*-arachidonoyl glycine (NAGly) and its precursor, the endocannabinoid *N*-arachidonoyl ethanolamide (Anandamide; AEA). Enzymatic pathways for endogenous AEA – to NAGly conversion were previously identified [13].

Neurons are known to use the endogenous cannabinoid system (eCBs) to communicate [1,2]. Walter *et al.* (2003) [3] reported the involvement of eCB signaling in recruiting microglia toward dying neurons *in vitro*. They demonstrated that pathological stimulation of neurons and microglia triggered microglial cell migration by engaging cannabinoid CB_2_ and abnormal cannabidiol (Abn-CBD) receptors. The Abn-CBD receptor is a prominent non-CB_1_/non-CB_2_ cannabinoid receptor that had been discriminated by means of various pharmacological and genetic tools but remained to be identified at the molecular level. It has been implicated in the modulation of microglial, endothelial and glioma cell migration, and a selection of cardiovascular responses [3-10]. We recently introduced *N*-arachidonoyl glycine (NAGly) signaling via GPR18 receptors as an important ‘new player’ in microglial-neuronal communication, providing a novel mechanism (both receptor and ligand) for directed migration and phenotypic switches in microglia [11,12]. NAGly is an endogenous, enzymatically oxygenated metabolite of the endocannabinoid *N*-arachidonoyl ethanolamide (Anandamide; AEA) (Figure 1) with two distinct biosynthetic pathways, one of which is fatty acid amide hydrolase (FAAH) dependent. NAGly is produced throughout the body, like AEA, but has no activity at either CB_1_ or CB_2_[13-16]. Instead, NAGly is known to act as a high affinity ligand for the G_i/o_-coupled GPCR GPR18 and a partial agonist of G_q/11_-coupled GPR92 receptors [17,18]. Moreover, NAGly is the most potent pro-migratory lipid with regard to BV-2 microglia reported to date. It is able to elicit a migratory response double that of 1 μM *N*-formyl-methionine-leucine-phenylalanine (fMLP), an established chemotactic peptide, at a concentration of 170 pM [11]. 

**Figure 2:**
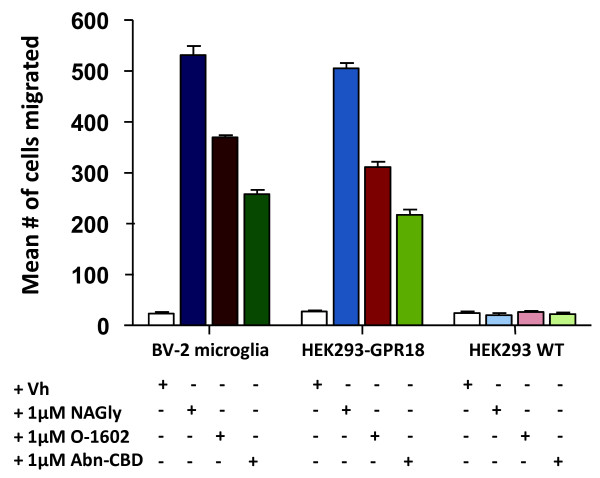
**Cell migration induced by NAGly, O-1602, Abn-CBD.** Cell migration in response to Vh (0.1% DMSO); 1 μM NAGly; 1 μM O-1602; 1 μM Abn-CBD in BV-2, HEK293-GPR18 and HEK293 WT cells. *n* = 3. This figure represents data previously published [11] and is intended to illustrate the context of the novel findings reported here.

Our published studies [11,12] support strongly two hypotheses; first, NAGly initiates directed microglial migration in the CNS through activation of GPR18, and second, GPR18 is the Abn-CBD receptor reported to be present in microglia: BV-2 microglia show GPR18 receptor immunoreactivity and qPCR demonstrates that primary microglia, likewise, express abundant GPR18 mRNA. The pharmacological profile characteristic of the G_i/o_-coupled Abn-CBD receptor includes activation by Abn-CBD and O-1602, which are inactive at CB_1_ and CB_2_, and antagonism by O-1918 and CBD [3,5-7,19]. Migration assays with wildtype HEK293 and HEK293 cells stably transfected with GPR18 support our hypothesis that NAGly is acting via G_i/o_-coupled GPR18 receptors expressed by BV-2 microglia (Figure 2; This figure represents data previously published [11] and is intended to illustrate the context of the novel findings reported here). Pertussis toxin (PTX) blocks NAGly’s effects in BV-2 microglia and HEK293-GPR18 cells. Abn-CBD and O-1602 drive migration in GPR18 transfected cells but not wildtype, suggesting that GPR18 is the receptor target for Abn-CBD (Figure 2). Additionally, the migration induced by NAGly, Abn-CBD and O-1602 in BV-2 microglia and HEK293-GPR18 cells is attenuated by O-1918 and CBD [11]. Here we present siRNA knockdown data in further support of the hypotheses that GPR18 mediates the migratory effects of NAGly in BV-2 microglia, and that GPR18 is the Abn-CBD receptor present in microglia. 

## Results

In order to test our hypothesis that GPR18 receptors mediate the promigratory signaling of NAGly, O-1602 and Abn-CBD we first sought to silence GPR18 in BV-2 microglial cells.

**Figure 3:**
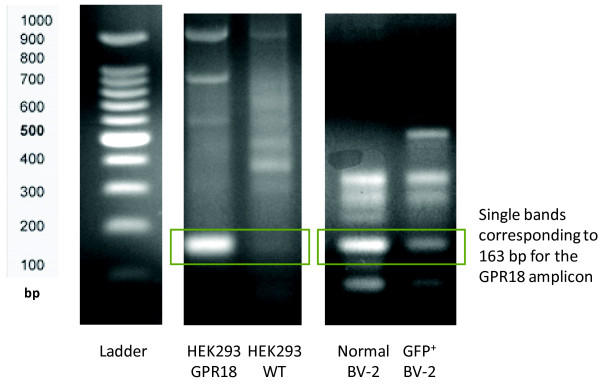
**GPR18 mRNA levels in siRNA transfected BV-2 microglia.** Gel electrophoresis of HEK293-GPR18, HEK293 WT, Normal BV-2 microglia and GFP^+^ GPR18 siRNA transfected BV-2 microglia RT-PCR products run on a 2% agarose gel.

**Figure 4:**
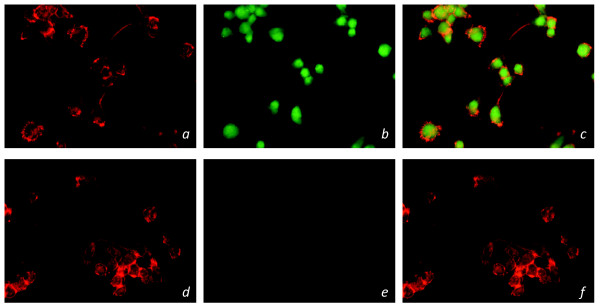
**Immunocytochemical staining of GPR18 receptors in siRNA transfected BV-2 microglia.** Immunofluorescent confocal microscopy was conducted using an antibody against the GPR18 C-terminus (1:150; green), phalloidin to label actin (1:40; red). Normal BV-2 microglia with: *a*) phalloidin *b*) GPR18 antibody *c*) phalloidin and GPR18 antibody. GFP^+^ GPR18 siRNA transfected BV-2 microglia with: *d*) phalloidin *e*) GPR18 antibody *f*) phalloidin and GPR18 antibody.

A GPR18-targetting siRNA pSUPER G418 GFP cDNA plasmid was created and transfected into BV-2 microglia. Successfully transfected GFP^+^ GPR18 siRNA BV-2 microglia were then sorted from non-transfected BV-2 cells via FACS analysis. Reverse-transcriptase PCR was employed to assess the knockdown of GPR18 mRNA in GFP^+^ BV-2 cells. HEK293 non-transfected, HEK293-GPR18 transfected and normal BV-2 cells were included as controls (Figure 3). Figure 3 shows the definitive band at 163 bp that coincides with the primer product for GPR18 mRNA, and indicates that GPR18 mRNA levels in GFP^+^ BV-2 microglia were successfully attenuated. Immunocytochemical staining of GPR18 receptors revealed a similar observation that in GFP^+^ BV-2 cells GPR18 receptor expression is substantially reduced (Figure 4).

Having verified effective knockdown of GPR18 in the GFP^+^ transfected BV-2 microglia, we next investigated the migration of these cells compared to normal BV-2 microglia.

**Figure 5:**
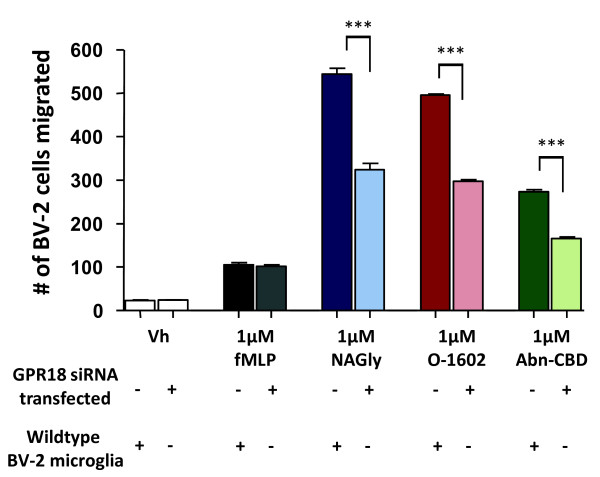
**Cell migration induced by NAGly, O-1602, Abn-CBD in wildtype BV-2 microglia (lefthand bars) and siRNA transfected BV-2 microglia (righthand bars).** Cell migration in response to Vh (0.1% DMSO); 1 μM fMLP; 1 μM NAGly; 1 μM O-1602; 1 μM Abn-CBD in Normal BV-2 microglia and GFP^+^ GPR18 siRNA transfected BV-2 microglia. *n* = 3; *** = P < 0.001, one-way ANOVA.

Migration induced by 1 μM concentrations of NAGly, O-1602 and Abn-CBD was significantly attenuated in GFP^+^ cells compared to control, whereas migration to Vh (0.1% DMSO) and fMLP remained unchanged (Figure 5; p < 0.001, one-way ANOVA). fMLP is a tripeptide chemoattractant released from both bacteria and damaged mitochondria [20,21], and activates two formyl peptide receptors, designated FPR and FPRL-1; silencing of GPR18 would be expected to have no effect on fMLP signaling. A control population of BV-2 cells transfected with the original pSUPER G418 GFP vector (i.e., the plasmid encoded GFP but not a GPR18 RNAi sequence) behaved no differently from wildtype BV-2 microglia. Our data provide definitive evidence that these compounds, characteristic of Abn-CBD receptor pharmacology, are acting via GPR18 in BV-2 microglia. 

## Discussion

For years well-documented evidence has supported the existence of additional cannabinoid receptors, other than CB_1_ and CB_2_, contributing to the system [5,6,22]. Our McHugh *et al.* (2010) study [11] was the first to demonstrate that the G_i/o_-coupled GPCR, GPR18, is one of these unknown receptors in that AEA and its metabolite NAGly exert potent control of central nervous system microglia. Since then we have further characterized GPR18 pharmacology via p44/42 mitogen activated kinase (MAP kinase) activation [12]. We proposed the working hypotheses: first, that NAGly initiates directed microglial migration in the CNS through activation of GPR18; and second, that GPR18 is the molecular identity of the Abn-CBD receptor present in microglia. Here, we provide definitive evidence in support of these. Namely, that NAGly, O-1602 and Abn-CBD – compounds characteristic of Abn-CBD receptor pharmacology – are acting via GPR18 in BV-2 microglia. Figure 5 shows the statistically significant and substantial attenuation of cell migration in GFP^+^ BV-2 microglia. The lack of a complete block of the NAGly, O-1602 and Abn-CBD effects may suggest an additional GPCR target for these ligands other than GPR18. However, it is important to note that achieving 100% siRNA knockdown efficiency is highly problematic, especially in cells where the gene in question is performing an essential function. The reduced but still detectable GPR18 amplicon band and immunocytochemical staining imply that enough GPR18 mRNA is being transcribed to allow these compounds to continue to signal. Future studies with GPR18 knockout animals will help clarify this.

An understanding of the expression, function, and regulation of the hitherto unidentified cannabinoid receptors such as GPR18; their molecular interactions with endogenous ligands; and how phytocannabinoids influence their signaling is vital if we are to comprehensively assess the function of the endogenous cannabinoid signaling system in human health and disease.

## Methods

### Cell culture

BV-2 cells (a gift from Dr. N. Stella; University of Washington, Seattle), an immortalized mouse microglial cell line, were grown in high glucose DMEM (Gibco, USA) with FBS (5%; J R Scientific, USA), penicillin (100 units/ml; Sigma, USA), streptomycin (100 μg/ml; Sigma, USA) and L-glutamine (0.292 mg/ml; Gibco, USA), and passaged every 4–5 days for a maximum of 30 passages. Twenty-four hours prior to experimentation, the culture media was replaced by serum-free high-glucose DMEM supplemented with penicillin (100 units/ml), and streptomycin (100 μg/ml). Test compounds were dissolved in DMSO to a final concentration of 0.1%

### siRNA knockdown of GPR18

Initially, custom double-stranded GPR18 Stealth RNAi™ siRNA primers were purchased from Invitrogen. Three primer pairs were ordered to maximize the probability of achieving successful GPR18 knockdown. The double-stranded siRNA pairs were introduced into BV-2 microglial cells in order to activate the cell’s RNAi pathway and interfere with the expression of GPR18 (see Additional file 1 for further details). The cells were evaluated for GPR18 silencing and transfection efficiency was determined to be less than 10%. Therefore we next employed the pSUPER vector system, which is designed specifically for the expression of short interfering RNA (siRNA).

Transfection of an exogenous siRNA can be problematic because the gene knockdown effect is only transient, particularly in rapidly dividing cells. One way of overcoming this challenge is to modify the siRNA in such a way as to allow it to be expressed by an appropriate vector, e.g., a plasmid. This is done by the introduction of a loop between the two strands, thus producing a single transcript, which can be processed into a functional siRNA. Such transcription cassettes typically use an RNA polymerase III promoter (e.g., H1), which directs the transcription of small nuclear RNAs (snRNAs) (H1 is the RNase component of human RNase P). The resulting siRNA transcript would then processed by the enzyme, Dicer.

To effect the silencing of murine GPR18, the pSUPER G418 GFP vector was used in concert with 3 pairs of custom oligonucleotides that contain a unique 19-nt sequence (the “N-19 target sequence”) derived from the mRNA transcript of the murine GPR18 gene. The N-19 target sequence, found using Ambion siRNA Target Finder (Applied Biosystems), corresponds to the sense strand of the pSUPER-generated siRNA, which in turn corresponds to a 19-nt sequence within the murine GPR18 mRNA.

#### GPR18 RNAi sequence 1

Sense GATCCCCtcacaaccagcttgatcttttTTCAAGAGAaaaa gatcaagctggttgtgaTTTTT

AntiSense agctAAAAAtcacaaccagcttgatcttttTCTCTTGAAaaaagatcaagctggttgtgaGGG

#### GPR18 RNAi sequence 2

Sense GATCCCCtggctcacacccagaggaattTTCAAGAGA aattcctctgggtgtgagccaTTTTT

AntiSense agctAAAAAtggctcacacccagaggaattTCTCTTGAAaattcctctgggtgtgagccaGGG

#### GPR18 RNAi sequence 3

Sense GATCCCCtcgcagccctagtcttctattTTCAAGAGAaatagaagactagggctgcgaTTTTT

AntiSense agctAAAAAtcgcagccctagtcttctattTCTCTTGAAaatagaagactagggctgcgaGGG

In the mechanism of RNAi, the antisense strand of the siRNA duplex hybridizes to this region of the mRNA to mediate cleavage of the molecule. These forward and reverse oligos were annealed and cloned into the vector, between the unique BgIII and HindIII enzyme sites. This positions the forward oligo at the correct position downstream from the H1 promoter’s TATA box to generate the desired siRNA duplex (see Additional file 1 for further details).

Lipofectamine 2000 (Invitrogen; USA) was employed to transfect BV-2 microglia with this custom plasmid (see Additional file 1 for further details). BV-2 microglia were then monitored for GFP fluorescence to indicate successful transfection and processing of the GPR18-containing pSUPER GFP G418 cDNA plasmid. Only BV-2 microglia transfected with GPR18 RNAi sequence 1 produced such GFP fluorescence. We tried to select the successfully transfected BV-2 microglia with G418 antibiotic in order to establish a stable cell line. This was unsuccessful; BV-2 microglia do not tolerate G418 antibiotic selection well. Therefore, using a fluorescence-activated cell sorting (FACS) Aria II, the GFP^+^ subpopulation of BV-2 microglia were aseptically sorted from the non-transfected GFP^-^ subpopulation based on the fluorescent characteristics of each cell. The FACS Aria II additionally discarded the <1% of dead BV-2 cells. Cells were allowed to recover for 3 h in serum-free DMEM before being used for experimentation. A control population of BV-2 microglia were similarly transfected with the original pSUPER G418 GFP vector (i.e., the plasmid encoded GFP but not a GPR18 RNAi sequence) and sorted via FACS.

### Reverse transcriptase PCR

Reverse-transcriptase PCR was employed to assess the knockdown of GPR18 mRNA in the GFP^+^ BV-2 cells. Standard PCR techniques were performed with optimized GPR18 primers that had been verified in BV-2 microglia and GPR18-transfected HEK293 cells as previously described [11].

### Immunocytochemistry

Briefly (see Additional file 1 for further details), cells were fixed with 3.7% paraformaldehyde, blocked, and stained as follows: polyclonal rabbit anti-C-terminal GPR18 (1:150) (generated in a previous study) [11] and Texas Red-conjugated phalloidin (1:40; Molecular Probes, Eugene, OR). Secondary IgG antibodies were FITC-conjugated donkey anti-rabbit (1:150; Jackson ImmunoResearch, USA). Images were acquired with a Nikon Eclipse TE2000-E confocal microscope (Nikon, USA).

### *In vitro* cell migration assays

Were performed using a modified 96-well Boyden chamber. BV-2 cells, 1×10^6^ cells/ml, were loaded into the upper wells; lower wells contained test compound. The chamber was incubated in a 5% CO_2_ atmosphere at 37°C for 3 h. Following incubation, migrated cells were stained with Diff-Quik® then counted in ten non-overlapping fields (×40) by multiple investigators. BV-2 cell migration in response to test compounds were expressed as the mean number of cells migrated.

### Analysis of data

All data are expressed as means ± s.e.mean and *n* = number of independent experiments. Statistical analyses were performed with GraphPad Prism 4.

## Abbreviations

Abn-CBD: Abnormal cannabidiol; ANOVA: Analysis of variance; AEA: *N*-arachidonoyl ethanolamine; CBD: Cannabidiol; CB_1_: Cannabinoid receptor 1; CB_2_: Cannabinoid receptor 2; CNS: Central nervous system; DMEM: Dulbecco’s Minimum Essential Medium; DMSO: Dimethyl sulphoxide; eCBS: Endogenous cannabinoid system; FAAH: Fatty acid amide hydrolase; FACS: Fluorescence activated cell sorting; FBS: Fetal bovine serum; fMLP: *N*-formyl-methionine-leucine-phenylalanine; FPR: Formyl peptide receptor 1; FPRL-1: Formyl peptide receptor-like 1; GFP: Green fluorescence protein; GPCR: G protein-coupled receptor; MAP kinase: Mitogen activated protein kinase; NAGly: *N*-arachidonoyl glycine; PTX: Pertussis toxin; O-1602: *Trans*-4- [3-methyl-6-(1-methylethenyl)-2-cyclohexen-1-yl]-5-methyl-1,3-benzenediol; O-1918: 1,3-dimethoxy-5-methyl-2- [(1R,6R)-3-methyl-6-(1-methylethenyl)-2-cyclohexen-1-yl)-benzene; RT-PCR: Reverse transcriptase polymerase chain reaction; siRNA: Short interfering messenger ribonucleic acid.

## Competing interests

The authors declare that they have no competing interests.

## Authors’ contributions

DM performed the cell culture procedures, siRNA procedures, immunocytochemistry imaging, cell migration studies; design and coordination of the studies; data interpretation; statistical analyses; and manuscript preparation. JW-M conducted siRNA procedures. JP conducted the PCR studies. HBB design and coordination of the studies, and manuscript preparation. All authors read and approved the final manuscript.

## Supplementary Material

Additional file 1 siRNA knockdown of GPR18 with Invitrogen custom siRNA primers. Click here for file
