# Supplementary material for: siRNA knockdown of GPR18 receptors in BV-2 microglia attenuates N-arachidonoyl glycine-induced cell migration
Source: J Mol Signal. 2012 Jul 26;7:10. doi: 10.1186/1750-2187-7-10 (PMC3493281; doi:10.1186/1750-2187-7-10)
Supplement: Additional file 1 — siRNA knockdown of GPR18 with Invitrogen custom siRNA primers. [file 1750-2187-7-10-S1.docx]

**Supplementary Methods**

**siRNA knockdown of GPR18 with Invitrogen custom siRNA primers**

Initially, custom double-stranded GPR18 Stealth RNAi™ siRNA primers were purchased from Invitrogen. Three primer pairs were ordered to maximize the probability of achieving successful GPR18 knockdown. The double-stranded siRNA pairs were introduced into BV-2 microglial cells in order to activate the cell’s RNAi pathway and interfere with the expression of GPR18.

*Oligonucleotide pair 1*

RNA: ACGUCUAAGCUGAAGCCCAAGGUCA

RNA: UGACCUUGGGCUUCAGCUUAGACGU

*Oligonucleotide pair 2*

RNA: UACUGCUCUACGAAGACCCAGACAA

RNA: UUGUCUGGGUCUUCGUAGAGCAGUA

*Oligonucleotide pair 3*

RNA: ACCUGCCUGAAGAUCUCCGACAUCA

RNA: UGAUGUCGGAGAUCUUCAGGCAGGU

Transfection procedure:

One day before transfection, BV-2 microglia were seeded in 6-well plates such that they reached ~ 50% confluency at the time of transfection in 500 μl of medium. For each well to be tranfected, RNAi duplex-Lipofectamine RNAiMAX complexes were prepared: first, 60 pmol of RNAi duplex was added to 250 μl Opti-MEM I reduced serum medium without serum and gently mixed; secondly, 20 μl of Lipofectamine RNAiMAX was diluted in 250 μl Opti-MEM medium and gently mixed; next, the diluted RNAi duplex was combined with the diluted Lipofectamine RNAiMAX and gently mixed before being incubated for 20 minutes at room temperature. Following this, the RNAi duplex-Lipofectamine RNAiMAX complexes were added to each well of cells and gently mixed by rocking. This gave a final volume of 1 ml and a final RNA concentration of 100 nM. Cells were incubated at 37°C in a CO_2_ incubator for 4 hours. Following this, the media was changed and the cells were allowed to recover for 24 hours in complete medium (DMEM 10% FBS).

The cells were evaluated for GPR18 silencing and transfection efficiency was determined to be less than 10%.

**siRNA knockdown of GPR18 with pSUPER GFP G418 siRNA vector**

The pSUPER vector system is designed specifically for the expression of short interfering RNA (siRNA). Transfection of an exogenous siRNA can be problematic because the gene knockdown effect is only transient, particularly in rapidly dividing cells. One way of overcoming this challenge is to modify the siRNA in such a way as to allow it to be expressed by an appropriate vector, e.g., a plasmid. This is done by the introduction of a loop between the two strands, thus producing a single transcript, which can be processed into a functional siRNA. Such transcription cassettes typically use an RNA polymerase III promoter (e.g., H1), which directs the transcription of small nuclear RNAs (snRNAs) (H1 is the RNase component of human RNase P). The resulting siRNA transcript would then processed by the enzyme, Dicer.

To effect the silencing of murine GPR18, the pSUPER G418 GFP vector was used in concert with 3 pairs of custom oligonucleotides that contain a unique 19-nt sequence (the “N-19 target sequence”) derived from the mRNA transcript of the murine GPR18 gene. The N-19 target sequence, found using Ambion siRNA Target Finder (Applied Biosystems), corresponds to the sense strand of the pSUPER-generated siRNA, which in turn corresponds to a 19-nt sequence within the murine GPR18 mRNA.

*GPR18 RNAi sequence 1*

Sense GATCCCCtcacaaccagcttgatcttttTTCAAGAGAaaaagatcaagctggttgtgaTTTTT

AntiSense agctAAAAAtcacaaccagcttgatcttttTCTCTTGAAaaaagatcaagctggttgtgaGGG

*GPR18 RNAi sequence 2*

Sense GATCCCCtggctcacacccagaggaattTTCAAGAGAaattcctctgggtgtgagccaTTTTT

AntiSense agctAAAAAtggctcacacccagaggaattTCTCTTGAAaattcctctgggtgtgagccaGGG

*GPR18 RNAi sequence 3*

Sense GATCCCCtcgcagccctagtcttctattTTCAAGAGAaatagaagactagggctgcgaTTTTT

AntiSenseagctAAAAAtcgcagccctagtcttctattTCTCTTGAAaatagaagactagggctgcgaGGG

In the mechanism of RNAi, the antisense strand of the siRNA duplex hybridizes to this region of the mRNA to mediate cleavage of the molecule. These forward and reverse oligos were annealed and cloned into the vector, between the unique BgIII and HindIII enzyme sites. This positions the forward oligo at the correct position downstream from the H1 promoter’s TATA box to generate the desired siRNA duplex.

Transfection procedure:

The forward and reverse strands of the oligos that contain the GPR18 siRNA-expressing sequence were annealed. The pSUPER vector was then linearized with BgIII and HindIII restriction enzymes. The annealed oligos were then cloned into the pSUPER GFP G418 vector and the vector transformed in bacteria. 10 colonies of bacteria were subsequently selected. The resulting plasmids from each colony were sequenced to ensure they retained the desired target sequence based on the GPR18 gene. The day before transfection, BV-2 microglia were seeded in 6-well plates such that they reached ~ 50% confluency on the day of transfection. 60 μl OptiMEM + 5 μl Superfect transfection reagent (Invotrgoen) + 2 μg cDNA plasmid were mixed gently together in a sterile eppendorf tube and allowed to sit for 10 mins at room temperature. BV-2 cells were washed with PBS and then OptiMEM. 350 μl OptiMEM was added to each eppendorf tube of Superfect/cDNA and mixed well. The 350 μl OptiMEM was aspirated off the BV-2 microglia and replaced with Superfect/cDNA. Cells were allowed to incubate at 37°C in CO_2_ incubator for 3 hours. Following this, the media was changed and the cells were allowed to recover for 24 hours in complete medium (DMEM 10% FBS). BV-2 microglia were then monitored for GFP fluorescence to indicate successful transfection and processing of the GPR18-containing pSUPER GFP G418 plasmid.

Only BV-2 microglia transfected with GPR18 RNAi sequence 1 produced such GFP fluorescence. We tried to select the successfully transfected BV-2 microglia with G418 antibiotic in order to establish a stable cell line. This was unsuccessful; BV-2 microglia do not tolerate G418 antibiotic selection well. Therefore, using a fluorescence-activated cell sorting (FACS) Aria II, the GFP^+^ subpopulation of BV-2 microglia were aseptically sorted from the non-transfected GFP^-^ subpopulation based on the fluorescent characteristics of each cell. The FACS Aria II additionally discarded the <1% of dead BV-2 cells. Cells were allowed to recover for 3 hours in serum-free DMEM before being used for experimentation.

**Immunocytochemistry**

Cytoskeleton buffer with sucrose (CBS) was prepared: 10 mM MES pH 6.1, 138 mM KCl, 3 mM MgCl, 2 mM EGTA, 0.32 M sucrose. A 3.7% paraformaldehyde solution was diluted from 37% stock with CBS. 0.5% and 0.1% Triton-X solutions were prepared with PBS. The media was removed from the cells and immediately replaced with 2 ml of 3.7% paraformaldehyde CBS and allowed to fix for 20 mins at room temperature. The 3.7 paraformaldehyde CBS solution was then removed and discarded. Cells were rinsed with 2 ml of PBS. The PBS was removed and discarded. Cells were then permeabilized in 2 ml 0.5% Triton-X PBS solution for 10 mins, while gently rotating on a shaker. The 0.5% Triton-X PBS was then removed and discarded. The cells were then rinsed (3x) with 2 ml 0.1% Triton-X PBS. Cells were blocked with 0.5 ml of Odyssey blocking buffer for 10 mins. Next, 250 µl of Texas Red-Phalloidin antibody solution (1:40; Molecular Probes, Eugene, OR; dissolved in Odyssey blocking buffer) was added and gently rotated on a shaker for 20 mins. The Texas Red-Phalloidin antibody solution was then removed and discarded. The cells were washed (3 x) with 2 ml of 0.1% Triton-X PBS and then rinsed with PBS. Next, 250 µl of FITC-conjugated donkey anti-rabbit (1:150; Jackson ImmunoResearch, USA; dissolved in Odyssey blocking buffer) was added and gently rotated on a shaker for 1 hour while protected from light. The FITC-conjugated donkey anti-rabbit solution was then removed and discarded. The cells were washed (3 x) with 2 ml of 0.1% Triton-X PBS and then rinsed with PBS. Images were acquired with a Nikon Eclipse TE2000-E confocal microscope (Nikon, USA).
